# Rapid Evaluation of the Xpert^®^ Xpress CoV-2 *plus* and Xpert^®^ Xpress CoV-2/Flu/RSV *plus* Tests

**DOI:** 10.3390/diagnostics13010034

**Published:** 2022-12-22

**Authors:** Lara Dominique Noble, Lesley Erica Scott, Riffat Munir, Mignon Du Plessis, Kim Steegen, Lucia Hans, Puleng Marokane, Pedro Da Silva, Wendy Susan Stevens

**Affiliations:** 1 WITS Diagnostic Innovation Hub, Faculty of Health Sciences, University of the Witwatersrand, Johannesburg 2000, South Africa; 2 Centre for Respiratory Diseases and Meningitis, National Institute for Communicable Diseases, Division of the National Health Laboratory Service, Johannesburg 2192, South Africa; 3 School of Pathology, Faculty of Health Sciences, University of the Witwatersrand, Johannesburg 2000, South Africa; 4 National Priority Programmes, National Health Laboratory Service, Johannesburg 2000, South Africa

**Keywords:** SARS-CoV-2, respiratory tests, Xpert^®^ Xpress CoV-2 *plus*, Xpert^®^, Xpress CoV-2/Flu/RSV *plus*, diagnostic evaluation, novel target

## Abstract

The Xpert^®^ Xpress SARS-CoV-2 and Xpert^®^ Xpress SARS-CoV-2/Flu/RSV tests were rapidly developed and widely used during the severe acute respiratory syndrome coronavirus 2 (SARS-CoV-2) pandemic. In response to emerging genetic variability, a new SARS-CoV-2 target (RNA-dependent RNA-polymerase) has been added to both tests: Xpert^®^ Xpress CoV-2 *plus* and Xpert^®^ Xpress CoV-2/Flu/RSV *plus* test. A rapid evaluation of both tests was performed in South Africa, using residual respiratory specimens. Residual respiratory specimens (n = 125) were used to evaluate the Xpert^®^ Xpress CoV-2 *plus* test and included 50 genotyped specimens. The Xpert^®^ Xpress CoV-2/Flu/RSV *plus* test was assessed using 45 genotyped SARS-CoV-2 specimens, 10 influenza A, 10 influenza B and 20 respiratory syncytial virus specimens. Results were compared to in-country standard-of-care tests. Genotyped specimens tested the performance of the test under pressure from circulating SARS-CoV-2 variants of concern. Reference material was included to assess the test limits and linearity. The Xpert^®^ Xpress CoV-2 *plus* test performance compared to reference results across residual respiratory specimens was good (positive percentage agreement (PPA) = 95.2%, negative percentage agreement (NPA) = 95.0%) The Xpert^®^ Xpress CoV-2/Flu/RSV *plus* test showed good performance across all residual respiratory specimens (PPA = 100%, NPA = 98.3%). All genotyped variants of concern were detected by both tests. The Xpert^®^ Xpress CoV-2 *plus* and Xpert^®^ Xpress CoV-2/Flu/RSV *plus* tests can be used to diagnose SARS-CoV-2, and to diagnose and differentiate SARS-CoV-2, influenza A, influenza B and respiratory syncytial virus, respectively. The NPA was lower than the recommended 99%, but was influenced by the low number of negative specimens tested. The variants of concern assessed did not affect test performance. It is recommended that sites perform their own assessments compared to in-country standard-of-care tests.

## 1. Introduction

Coronavirus infectious disease of 2019 (COVID-19), which is caused by the severe acute respiratory syndrome coronavirus 2 (SARS-CoV-2) [1], was first detected in Wuhan, China, in December 2019. Even before the disease was deemed a pandemic by the World Health Organization (WHO) on 11 March 2020 [2], companies rapidly began to address the need for molecular diagnostics. Cepheid^®^ (Sunnyvale, CA, USA) launched the Xpert^®^ Xpress SARS-CoV-2 (Xpert^®^ Xpress SARS-CoV-2) in March 2020, with the United States of America Food and Drug Administration (FDA) Emergency Use Authorisation (EUA) received on 20 March 2020 [3]. South Africa leveraged the GeneXpert (Cepheid^®^) footprint, in place for tuberculosis testing since 2011 [4], as one of the testing platforms, with a rapid, in-house evaluation performed to show the test was fit for purpose for local use (unpublished data).

The Xpert^®^ Xpress SARS-CoV-2 test targets the SARS-CoV-2 specific nucleocapsid region two (N2) and pan sarbecovirus envelope (E) genes, and includes internal processing controls. This test has been widely evaluated [5,6,7,8,9,10,11] and used globally throughput the pandemic. However, with the emergence of SARS-CoV-2 variants [12,13,14,15,16,17,18,19,20,21] and concerns regarding N2-gene target failure (NGTF) [22,23,24,25], a third SARS-CoV-2 specific target, RNA-dependent RNA polymerase (RdRp), and N2-gene probe redundancy were added to the test [25], now named Xpert^®^ Xpress CoV-2 *plus* (FDA-EUA 10 May 2022 [3]). The RdRp target was included in the initial test design [10], but only recently incorporated for diagnostic purposes [25,26].

Furthermore, as the pandemic waned and other respiratory infections once more became prevalent, it became useful to rapidly differentiate between common respiratory viruses (influenza A (Flu A), influenza B (Flu B), respiratory syncytial virus (RSV)) and SARS-CoV-2, as disease symptoms are similar. Cepheid^®^ previously added the SARS-CoV-2 E- and N2-gene targets to their existing Xpert^®^ Xpress Flu/RSV test (read in a single channel without differentiation between gene targets), and this test has been successfully evaluated elsewhere [27,28,29,30,31,32], achieving FDA-EUA on 24 September 2020 [3]. This Xpert^®^ Xpress SARS-CoV-2/Flu/RSV test allows the detection and differentiation of SARS-CoV-2, Flu A, Flu B and RSV from a single nasopharyngeal or anterior nasal swab. Cepheid has now included the RdRp target in this test: Xpert^®^ Xpress CoV-2/Flu/RSV *plus* (FDA-EUA 10 September 2021 [3]).

The design and performance of these updated assays (research use only (RUO) format) in the presence of NGTF and emerging VOC has been well-described previously [25]. This study now details the rapid evaluation of both the Xpert^®^ Xpress CoV-2 *plus* and Xpert^®^ Xpress CoV2/Flu/RSV *plus* tests in South Africa. Residual clinical specimens, including VOC across all local COVID-19 waves, and reference materials were used, and the performance of both tests is described.

## 2. Materials and Methods

### 2.1. Overview

**Figure 1:**
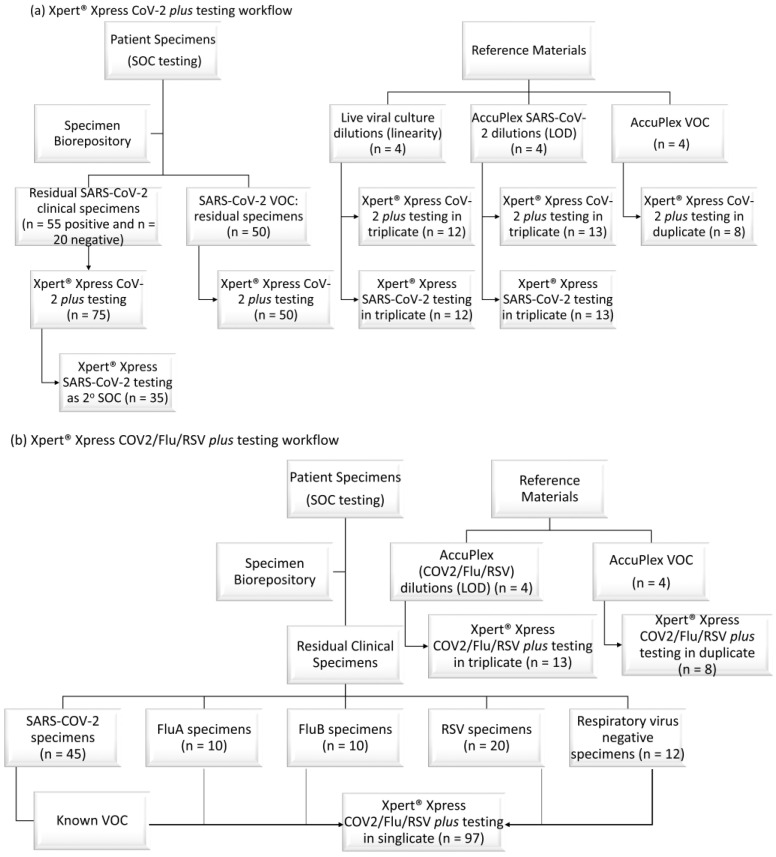
(**a**) Xpert^®^ Xpress CoV-2 *plus* workflow and (**b**) Xpert^®^ Xpress CoV-2/Flu/RSV *plus* workflow. 2^o^: secondary; LOD: limit of detection; n: number; SOC: standard of care; VOC: SARS-CoV-2 variants of concern.

The Xpert^®^ Xpress CoV-2 *plus* and Xpert^®^ Xpress CoV-2/Flu/RSV *plus* test (both Cepheid^®^) were rapidly evaluated using residual patient specimens. Specimen selection and workflow are shown in Figure 1a (Xpert^®^ Xpress CoV-2 *plus*) and 1b (Xpert^®^ Xpress CoV-2/Flu/RSV *plus*), respectively. The Xpert^®^ Xpress CoV-2 *plus* test was compared to standard-of-care (SOC) results and the Xpert^®^ Xpress SARS-CoV-2 test, while the Xpert^®^ Xpress CoV-2/Flu/RSV *plus* test was compared only to SOC results. In addition to performance against the reference results, the potential impact of variants of concern (VOC) on the tests’ analytical accuracy was evaluated. Specimens were selected across the four major waves in South Africa (Wildtype (Wuhan with D614G): April–July 2020; Beta: November 2020–January 2021; Alpha/Delta: May–July 2021; Omicron: October 2021–April 2022). Limited reference material was also included in the study, and is described below.

### 2.2. Ethics

Ethics approval for this evaluation was obtained from the University of the Witwatersrand Human Research Ethics Committee Medical (WITS HREC, Johannesburg, Gauteng, South Africa) as an amendment to the approval to access residual clinical specimens for SARS-CoV-2 test testing: M1911201.

### 2.3. Xpert^®^ Xpress CoV-2 plus Specimen Selection

Residual respiratory clinical specimens (n = 75), collected according to standard of care (SOC) protocols, were obtained from the National Health Laboratory Service (NHLS) and the National Institute of Communicable Diseases (NICD). Specimens were selected across local SARS-CoV-2 waves (as described above) and based on SOC SARS-CoV-2 results, and were stored at −80 °C until testing. The SARS-CoV-2 specimens used for the Xpert^®^ Xpress CoV-2 *plus* evaluation were supplied in a variety of media, including but not limited to universal transport medium (UTM), saline and phosphate-buffered saline (PBS). A limited number of SARS-CoV-2 negative specimens (n = 20) were included. Results were compared to two SOC comparators: cobas^®^ SARS-CoV-2 (cobas; Roche Molecular, Pleasanton, CA, USA) and TaqMan™ TaqPath COVID-19 (TaqPath; Thermo Fisher Scientific, Waltham, MA, USA). A limited number of specimens (n = 35) were further compared to the Xpert^®^ Xpress SARS-CoV-2 test currently in use in the NHLS in order to ensure non-inferiority of the Xpert^®^ Xpress CoV-2 *plus* test, which is necessary for potential implementation into national programmes. Specimen wave was noted as an indicator of likely VOC, but the VOC of these specimens was not confirmed.

The potential impact of circulating SARS-CoV-2 VOC on test performance was assessed using an additional 50 genotyped residual patient specimens. These specimens were selected by COVID-19 wave and were previously genotyped by the AllPlex SARS-CoV-2 Variants I and II and NovaPlex SARS-CoV-2 Variants V tests (all Seegene, Seoul, Republic of Korea) and/or TaqMan SARS-CoV-2 Mutation Panels (Thermo Fisher Scientific, Waltham, MA, USA) and/or MassARRAY SARS-CoV-2 Variant Panel v3 (RUO) (MassARRAY; Agena Biosciences, San Diego, CA, USA), or by next generation sequencing performed at the NICD. In addition, AccuPlex SARS-CoV-2 variant reference material (LGC SeraCare, Milford, MA, USA) was selected from the AccuPlex SARS-CoV-2 Variants Panel 1 (Wuhan, Alpha, Beta and Gamma) and AccuPlex SARS-CoV-2 Variants Panel 2 (Delta) were used to assess the performance of the test on VOC material. Briefly, AccuPlex material (5000 copies per millilitre (cp/mL)) was diluted to 500 cp/mL with molecular grade water (Sigma Aldrich, St. Louis, MO, United States) and each was tested twice. Omicron reference material was not available at the time of this evaluation.

The reported limit of detection (LOD) of the test (SARS-CoV-2 E-gene = 70 cp/mL, N2-gene = 403 cp/mL and RdRp = 200 cp/mL [33]) and test precision in the low viral burden range (<1000 cp/mL) were assessed using AccuPlex SARS-CoV-2 (LGC SeraCare, Milford, MA, USA) Wildtype (Wuhan) reference material. Specimens were diluted using molecular grade, nuclease-free water to 100, 250, 500 and 1000 cp/mL, and tested in triplicate by both the Xpert^®^ Xpress CoV-2 *plus* and Xpert^®^ Xpress SARS-CoV-2 tests, with a single undiluted (5000 cp/mL) specimen also processed (total = 13 tests). Wildtype SARS-CoV-2 culture material dilutions (at concentrations of approximately log 2.5, log 3.7, log 4.2 and log 4.7 cp/mL), prepared and eluted according to previously described protocols [34], were tested on the Xpert^®^ Xpress CoV-2 *plus* test in triplicate to assess the precision of the test across the range.

### 2.4. Xpert^®^ Xpress CoV-2/Flu/RSV plus Test Evaluation

Residual respiratory clinical specimens (n = 97), collected and tested according to standard of care (SOC) protocols, were obtained from the NICD and the NHLS. Specimens were selected based on SOC results and were stored at −80 °C until testing. The SARS-CoV-2 specimens used for the Xpert^®^ Xpress CoV-2-Flu-RSV *plus* evaluation were supplied in a variety of media (as described above), while the Flu and RSV specimens were collected in viral transport medium. In total, 45 SARS-CoV-2, 10 FluA, 10 FluB, 10 RSV A, 10 RSV B and 12 respiratory-virus negative specimens were tested. SOC testing was performed on multiple tests (TaqMan™ TaqPath COVID-19; AllPlex™ SARS-CoV-2 [Seegene, Seoul, Republic of Korea]; Xpert^®^ Xpress SARS-CoV-2; and cobas^®^ SARS-CoV-2), and on the AllPlex™ SARS-CoV-2/FluA/FluB/RSV (Seegene, Seoul, Republic of Korea). The last test allows differentiation of both Flu A and B, and RSV A and B subtypes. Xpert^®^ Xpress CoV-2/Flu/RSV *plus* results were compared to SOC results. In addition, the reported LOD of the test (SARS-CoV-2 = 138 cp/mL, Flu A(2019) = 0.007 TCID50/mL, Flu B(2019) = 12.9 CEID50/mL and RSV(2016) = 0.33 TCID50/mL [35]) and test precision in the low viral burden range (<1000 cp/mL) were assessed using AccuPlex SARS-CoV-2/FluA/FluB/RSV (LGC SeraCare, Milford, MA, USA) reference material diluted to 100, 250, 500 and 1000 cp/mL and tested in triplicate, with one undiluted specimen (5000 cp/mL) also tested (total = 13 tests). All SARS-CoV-2 specimens included (n = 45) were previously assessed for VOC type as described above, allowing assessment of the impact of VOC on the test. Omicron BA.4 specimens were not included due to limited specimen volume. The AccuPlex SARS-CoV-2 variant reference material (n = 5; LGC SeraCare) described above was also tested in duplicate on this test.

### 2.5. Xpert^®^ Test Overview

Xpert^®^ Xpress SARS-CoV-2, Xpert^®^ Xpress CoV-2 *plus* and Xpert^®^ Xpress CoV-2/Flu/RSV *plus* test testing were performed according to the manufacturer’s instructions for use (IFU) [33,35,36]. Briefly, 300 µL specimen was loaded into the relevant cartridge, the cartridge was sealed and loaded into a GeneXpert IV instrument for testing with the Xpert^®^ Xpress SARS-CoV-2, Xpert^®^ Xpress CoV-2 *plus* or Xpert^®^ Xpress CoV-2/Flu/RSV *plus* test definition file (ADF) respectively. Both 6-colour and 10-colour GeneXpert instruments were used for this study. The cartridges include a sample processing control (SPC) and a probe check control (PCC) to ensure the quality of the specimen (correct collection and processing, no PCR inhibitors, quality of PCR reagents) and the reagents (fluorescence check) respectively. Results are automatically generated by the GeneXpert™ DX System Version 6.2 software. While these tests report qualitative results, cycle threshold (Ct) values were available and were used for this analysis.

### 2.6. Data Analysis

Data were assessed using STATA SE 14.2 for Windows (StataCorp LP, College Station, TX, USA) and MedCalc^®^ Version 20.114 (MedCalc Software Ltd., Ostend, Belgium) software packages, with raw data being captured in Microsoft^®^ Excel (Redmond, WA, USA). The positive percentage agreement (PPA), negative percentage agreement (NPA) [37], agreement (Cohen kappa [38,39]) and precision compared to SOC (both tests) and Xpert^®^ Xpress SARS-CoV-2 (Xpert^®^ Xpress CoV-2 *plus* only) were calculated; 95 percent confidence intervals (95% CI) were included. Errors and invalid specimens were noted, and these results were excluded from the accuracy analysis.

## 3. Results

### 3.1. Xpert^®^ Xpress CoV-2 plus Evaluation

#### 3.1.1. Xpert^®^ Xpress CoV-2 *plus* Specimen Description

A total of 105 SARS-CoV-2 positive specimens and 20 SARS-CoV-2 negative specimens were tested using the Xpert^®^ Xpress CoV-2 *plus* test. Seventy-one of these had cobas^®^ SOC results, with a mean cobas^®^ Ct of 27.9 (range: 16.0, 36.9) for the E-gene and 27.1 (range: 14.5, 35.6) for the ORF1a gene. The other 34 SARS-CoV-2 positive specimens had TaqPath SOC results, with mean TaqPath Ct of 23.1 (range: 14.3, 32.0) for the ORF1ab gene, 23.4 (range: 17.7, 32.1) for the N-gene and 23.7 (range: 18.6, 29.8) for the S-gene. The specimen subset further tested on the Xpert^®^ Xpress SARS-CoV-2 test (n = 30 positive specimens) had a SOC ORF1ab mean Ct of 26.9 (range: 14.6, 35.6). The SARS-CoV-2 VOC set (n = 50) had a mean SOC ORF1ab Ct of 24.0 (range: 14.3, 32.0). Complete data is available in Supplementary Table S1.

#### 3.1.2. Xpert^®^ Xpress CoV-2 *plus* Accuracy Analysis

Accuracy was determined using clinically relevant specimens (n = 157), including 125 residual clinical specimens, 12 viral culture specimens and 20 AccuPlex specimens (Table 1). Only six errors (6/157 tests; 3.8% error rate) were observed and were excluded from accuracy analysis. No difference in performance was observed between the 6-colour and 10-colour GeneXpert Dx platforms.

The test performance across all specimens (n = 157) when compared to SOC or expected reference material results gave PPA of 96.4%, NPA of 95.0% and overall agreement of 0.8416. Similarly, the test performance across only residual clinical specimens (n = 125) compared to SOC gave PPA of 95.2%, NPA of 95.0% and overall agreement of 0.8348. There were a total of six discordant SARS-CoV-2 specimens compared to the SOC test (Table 2). One cobas^®^ SARS-CoV-2 (SOC) negative specimen (specimen 52) was positive by both the Xpert^®^ Xpress SARS-CoV-2 and Xpert^®^ Xpress CoV-2 *plus* tests, but negative on Xpert^®^ Xpress CoV-2 *plus* repeat testing. Five cobas^®^ positive specimens (specimens 110, 116, 119, 122, 125) were negative by Xpert^®^ Xpress CoV-2 *plus*, of which two were also negative by Xpert^®^ Xpress SARS-CoV-2. The Xpert^®^ Xpress CoV-2 *plus* test showed 100% agreement with the TaqPath test in 30 positive specimens tested.

The Xpert^®^ Xpress SARS-CoV-2 test was not used as SOC for the residual specimens. However, this test is used within the national testing program and thus could be considered as a SOC test. When compared to the Xpert^®^ Xpress SARS-CoV-2 test (n = 55 (30 SARS-CoV-2 SOC positive and 5 SARS-CoV-2 SOC negative residual patient specimens, and 20 AccuPlex SARS-CoV-2 positive specimens), PPA remained acceptable at 98.0%, with NPA decreasing to 87.5%. This was, however, influenced by the low number of negative specimens assessed (n = 7/55) and in reality reflects only one false negative specimen (specimen 119) with high Ct by the Xpert^®^ Xpress SARS-CoV-2 test (E = 38.4 N2 = 41.4). Similarly, only one false positive (specimen 114), compared to Xpert^®^ Xpress SARS-CoV-2, had high Ct by the Xpert^®^ Xpress CoV-2 *plus* test (E = 37.5, N2 = 42.3, RdRp = 0.0) and by SOC testing. Agreement remained very good at 0.8363. Discordant specimens are shown in Table 2.

Performance of the Xpert^®^ Xpress CoV-2 *plus* test across variants (Table S1) was evaluated using genotyped specimens (n = 50) and AccuPlex SARS-CoV-2 reference material diluted to 500 cp/mL (n = 11). The test detected 100% of specimens, indicating no impact on the test performance across all VOC to date at the time of the study, including Alpha, Beta, Delta, Omicron BA.1 and BA.4 specimens.

#### 3.1.3. Xpert^®^ Xpress CoV-2 *plus* Precision Analysis

**Figure 2:**
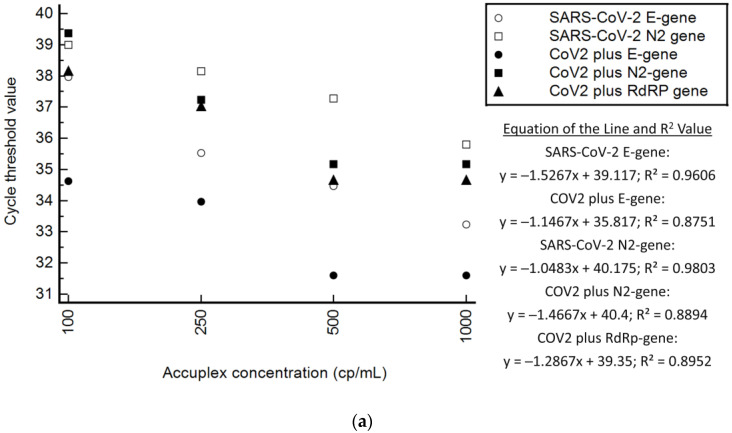

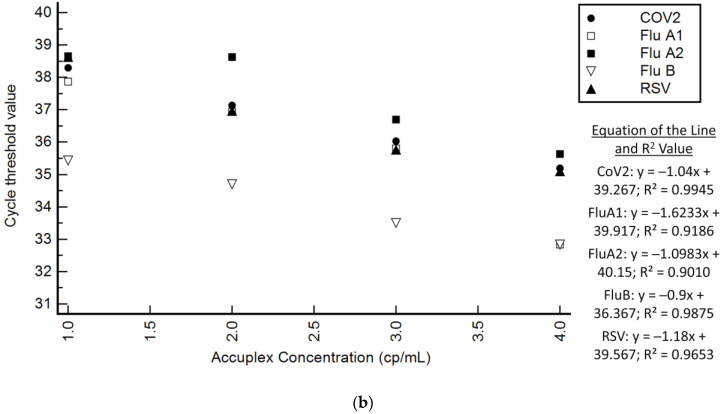
Regression analysis of the Xpert^®^ Xpress SARS-CoV-2, Xpert^®^ Xpress CoV-2 *plus* and Xpert^®^ Xpress CoV-2/Flu/RSV tests, including the equation of the line and R^2^ values. (**a**) Xpert^®^ Xpress SARS-CoV-2 and Xpert^®^ Xpress CoV-2 *plus*; (**b**) Xpert^®^ Xpress CoV-2/Flu/RSV *plus*.

AccuPlex reference material (Wildtype—Wuhan) was detected across all dilutions from 100 cp/mL to 1000 cp/mL by both the Xpert^®^ Xpress SARS-CoV-2 and Xpert^®^ Xpress CoV-2 *plus* tests for all gene targets (Table 3). Variability was observed for the N2 target at 250 cp/mL for the Xpert^®^ Xpress SARS-CoV-2 test, with one negative result, while all other targets were robustly detected by both tests. The E-gene target was detected at a lower Ct for Xpert^®^ Xpress CoV-2 *plus* test (mean Ct = 33.29) than for the Xpert^®^ Xpress SARS-CoV-2 test (mean Ct = 35.30). The Xpert^®^ Xpress CoV-2 *plus* N2-gene (mean Ct = 37.06) and RdRp (mean Ct = 36.50) targets were detected at similar Ct to the Xpert^®^ Xpress SARS-CoV-2 test N2-gene target (mean Ct = 37.55). The mean %CV across the tests (Xpert^®^ Xpress CoV-2 *plus*: E-gene = 0.60, N2-gene = 1.31, RdRp-gene = 1.03; Xpert^®^ Xpress SARS-CoV-2: E-gene = 3.11, N2-gene = 1.05) was consistently <5% (Table 3). The R^2^ values (Figure 2a) were slightly lower for the Xpert^®^ Xpress CoV-2 *plus* test (E-gene = 0.88, N2-gene = 0.89, RdRp = 0.90) compared to the R^2^ values for the Xpert^®^ Xpress SARS-CoV-2 test (E-gene = 0.96, N2-gene = 0.98).

### 3.2. Xpert^®^ Xpress CoV-2/Flu/RSV plus Evaluation

#### 3.2.1. Xpert^®^ Xpress CoV-2/Flu/RSV *plus* Specimen Description

The 45 SARS-CoV-2 specimens used for the Xpert^®^ Xpress CoV-2/Flu/RSV *plus* evaluation were tested on a number of SOC tests (cobas^®^, Xpert, TaqPath, AllPlex) and SOC Ct were thus reported by gene target rather than by test. The SARS-CoV-2 N-gene target is common to the majority of tests (71% results tested on Xpert^®^ Xpress SARS-CoV-2, TaqPath or AllPlex tests), with 32 specimens reporting a mean Ct of 24.9 (range: 16.1, 34.6), which is reflective of the overall test Ct values. In addition, 23 results included the S-gene target (TaqPath or AllPlex) with a mean Ct of 23.7 (range: 16.1, 34.6), 20 results included the ORF1ab gene (cobas^®^ or TaqPath) with a mean Ct of 24.5 (range: 17.0, 29.9), 15 results included the RdRp gene (AllPlex) with a mean Ct of 24.7 (range 15.1, 34.5) and 12 results included the E-gene (cobas^®^ or Xpert^®^ Xpress SARS-CoV-2) with a mean Ct of 25.0 (range: 14.9, 29.2). A limited number of SARS-CoV-2 negative specimens (n = 12) were included, with the 40 respiratory specimens also listed as SARS-CoV-2 negative for the purposes of the study.

The respiratory specimens were all tested by AllPlex™ SARS-CoV-2/FluA/FluB/RSV and stratified by virus. The SOC mean Ct was 27.6 (range: 22.3, 34.8) for the Flu A specimens (n = 10), 28.1 (range: 18.5, 35.2) for the Flu B specimens (n = 10) and 25.5 (range: 18.6, 36.0) for the RSV specimens (n = 10 RSV A and n = 10 RSV B). The RSV subtype is not differentiated on the Xpert^®^ Xpress CoV-2/Flu/RSV test and the SOC Ct was thus reported as a single result.

#### 3.2.2. Xpert^®^ Xpress CoV-2/Flu/RSV *plus* Accuracy Analysis

Accuracy was determined using residual clinical specimens (n = 97) and reference material. Only one error due to cartridge failure was observed (1/118 tests; 0.85% error rate) and was excluded from accuracy analysis. No difference in performance was observed between the 6-colour and 10-colour GeneXpert Dx platforms. Complete results are shown in Supplementary Table S2.

When assessed across all disease targets (Table 4), the test showed excellent overall PPA of 100% and very good agreement (0.8972). NPA was lower than generally acceptable at 83.3%, but was negatively influenced by the low number of negative specimens (n = 12). However, when each disease target was assessed individually, using the other specimens as target-disease negative, the overall accuracy improved across both clinically relevant specimens (n = 452, PPA: 97.9%, NPA: 98.4%) and residual patient specimens (n = 368, PPA: 100%, NPA: 98.3%). Discordant specimens are shown in Table 2. There were two SARS-CoV-2 false positive results (specimens 82 and 83) compared to cobas^®^ SARS-CoV-2, both with high Xpert^®^ Xpress CoV-2 *plus* Ct of 38.5 and 39.8, respectively. There were a further three RSV-positive specimens that were also positive for SARS-CoV-2 by the Xpert^®^ Xpress CoV-2/Flu/RSV *plus* test (specimens 27, 30 and 34). All had high Xpert^®^ Xpress SARS-CoV-2 Ct values (36.1, 40.9 and 43.5). Confirmatory SARS-CoV-2 testing by an alternate test was not possible due to limited specimen volume. No Flu A, Flu B or RSV specimens were misclassified amongst the residual patient specimens, with only one AccuPlex dilution (100 cp/mL) reporting a RSV negative result with a high Ct of 39.7. Two AccuPlex specimens were negative for Flu A2 (100 cp/mL, Ct = 0.0; 250 cp/mL, Ct = 40.6), but positive for Flu A1, with overall positive Flu A results reported.

Performance of the Xpert^®^ Xpress CoV-2/Flu/RSV *plus* test across SARS-CoV-2 VOC was possible as the 45 SARS-CoV-2 specimens were previously genotyped specimens. The test detected 100% of specimens (Table S2), including the five AccuPlex specimens tested in duplicate, indicating no impact on the test across all VOC to date, including Alpha, Beta, Gamma, Delta and Omicron VOC. This analysis was performed on Omicron BA.1 specimens and not further sub-variants (BA.2–5).

#### 3.2.3. Xpert^®^ Xpress CoV-2/Flu/RSV *plus* Precision Analysis

AccuPlex reference material (SARS-CoV-2, Flu A, Flu B, RSV) was detected by the Xpert^®^ Xpress CoV-2/Flu/RSV *plus* test across all dilutions from 100 cp/mL to 1000 cp/mL for all gene targets (Table 5). Variability was observed for the Flu A2 target at 100 cp/mL (not detected) and 250 cp/mL (Ct = 40.6, above the acceptable limit), and for RSV at 100 cp/mL (Ct 39.7, above acceptable limit). SD and %CV for all disease targets were acceptable, with an overall mean SD of 0.41 (range: 0.21, 0.66) and an overall mean %CV of 1.13 (range 0.56, 1.71). Linearity (Figure 2b) in the low viral burden range (<1000 cp/mL) was good, with R^2^ across the target viruses >0.9.

## 4. Discussion

The evaluation of novel or redesigned diagnostics is important to ensure ongoing testing of expected quality, ultimately impacting patient care. This study describes a rapid evaluation of the Xpert^®^ Xpress CoV-2 *plus* and Xpert^®^ Xpress CoV-2/Flu/RSV *plus* tests using residual clinical specimens and compared to SOC results. There is currently limited published information on the new iterations of these tests [25,26], with this study adding further information to the knowledge base, including testing of the Beta variant, which was not previously widely evaluated with these assays [25]. While the positive percentage agreement (sensitivity) of the tests is in line with the World Health Organization recommendations [40], the negative percentage agreement (specificity) is lower than ideal. This is due to a low number of negative specimens being tested, as discussed further below.

The Xpert^®^ Xpress CoV-2 *plus* test showed acceptable performance compared to SOC test results (cobas^®^ SARS-CoV-2 and TaqMan^®^ TaqPath COVID-19) for testing residual patient specimens. The PPA of the test (96.4%) is in line with that described for the Xpert^®^ Xpress SARS-CoV-2 test in a recent meta-analysis (97.0%) [11], with very good agreement [41] of 84.16%. The lower NPA (95.0% compared to 97.0%) [11] was skewed by the low numbers of negative specimens included in this analysis and in reality was only one false positive result (1/20), with a repeat testing being negative. This, and the false negative results (cobas^®^ SARS-CoV-2 mean Ct of 36.6 for the E-gene target and 34.9 for the ORF1a target), are believed to be near the LOD for the cobas^®^ SARS-CoV-2, Xpert^®^ Xpress SARS-CoV-2 and Xpert^®^ Xpress CoV-2 *plus* tests, leading to increased variability in results. A brief report [42] describing good agreement between the cobas^®^ SARS-CoV-2 and the Xpert^®^ Xpress SARS-CoV-2 tests at higher Ct values had similar results, with 1/35 specimens testing positive by cobas^®^ SARS-CoV-2 and negative by Xpert^®^ Xpress SARS-CoV-2. The results of the new test also concur with the original evaluations of the Xpert^®^ Xpress SARS-CoV-2 test compared to seven SOC tests (99.5% PPA and 95.8% NPA) [10], although the agreement is lower than that described in comparison to the cobas^®^ SARS-CoV-2 test (89% as opposed to 99%) [5]. The latter study included 59% negative specimens that were tested fresh, whereas only 16% of specimens included in our study were negative. Lastly, the Xpert^®^ Xpress CoV-2 *plus* test also showed acceptable PPA (98.0%) when compared to the Xpert^®^ Xpress SARS-CoV-2 test. This is similar to the perfect agreement between the Xpert^®^ Xpress SARS-CoV-2 and Xpert^®^ Xpress CoV2 tests on 50 positive specimens, including VOC and NGTF specimens [25]. The decreased NPA (85.7%) observed is believed to be an artefact of the low number of negative specimens assessed (12%) and in reality reflects only one false negative specimen with high Ct values. Agreement compared to the Xpert^®^ Xpress SARS-CoV-2 test was also very good [41] at >80%. Precision of the Xpert^®^ Xpress CoV-2 *plus* and Xpert^®^ Xpress SARS-CoV-2 tests were similar, with low SD (<1.2 Ct) and %CV (<3.2%) down to 100 cp/mL AccuPlex SARS-CoV-2. While larger studies with increased numbers of negative specimens are recommended, it is likely that the Xpert^®^ Xpress CoV-2 *plus* test can be used equivalently to the Xpert^®^ Xpress SARS-CoV-2 test. Few errors were noted for either test and no difference in results was observed between the 6-colour and 10-colour instruments. Additionally, the Xpert^®^ Xpress CoV-2 *plus* test provides results more rapidly (~31 min) than the Xpert^®^ Xpress SARS-CoV-2 test (~48 min).

It is of interest that the early evaluation of the Xpert^®^ Xpress SARS-CoV-2 test by Loeffelholz and colleagues [10], which was performed on the Xpert^®^ Xpress SARS-CoV-2 RUO tests and included the RdRp target that was not included in the first Xpert^®^ Xpress SARS-CoV-2 EUA test, noted that the presence of E, N2 and RdRp was consistent amongst concordant positive results, with discordant positive specimens usually showing only the E-gene or N2-gene targets. The sole specimen negative by cobas^®^ SARS-CoV-2 and detected by Xpert^®^ Xpress SARS-CoV-2 (E-gene, N2-gene positive) and Xpert^®^ Xpress CoV-2 *plus* (E-gene, N2-gene and RdRp-gene positive) showed all gene targets, including RdRp. Similarly, in our study, the E-gene target showed lower Ct values than the N2-gene and RdRp-gene targets, with the latter targets showing similar Ct throughout. Only one specimen (positive by cobas^®^ SARS-CoV-2 and negative by Xpert^®^ Xpress SARS-CoV-2) was Xpert^®^ Xpress CoV-2 *plus* E-gene and N2-gene positive and RdRp-gene negative, so it is not possible to comment on the decreased sensitivity of RdRp observed in the earlier study [10]. The RdRp-gene target has not been modified since the original study, but was recently noted to be more sensitive than the N2-gene target [25]. A recent study also showed that Xpert^®^ Xpress SARS-CoV-2 N2-gene only positive specimens were also positive by digital drop PCR, indicating a low viral burden [43]. However, in this study no E-gene dropout was observed, with five specimens SARS-CoV-2 positive by E-gene only, with high cobas^®^ Ct values.

The Xpert^®^ Xpress CoV-2/Flu/RSV *plus* test showed comparable performance to the SOC tests, with an overall PPA of 100% and NPA of 98.3%. Individually, the Flu A, Flu B and RSV targets reported 100% agreement, while the SARS-CoV-2 target had 100% PPA and 90.4% NPA. This NPA is lower than the NPA reported in a recent multicentre evaluation of the original Xpert^®^ Xpress SARS-CoV-2/Flu/RSV test (98.7%) against a range of tests [32], and against the Xpert^®^ Xpress SARS-CoV-2 (100%) and Xpert^®^ Xpress Flu/RSV (>99.5%) tests in a separate study [31]. An important, recent preprint [26] evaluated the Xpert^®^ Xpress CoV-2/Flu/RSV *plus* test using the residual specimens from an evaluation of the Xpert^®^ Xpress SARS-CoV-2/Flu/RSV [30], with 100% agreement for SARS-CoV-2, Flu A and RSV, and 99.4% for Flu B [26]. The study included 50 clinical specimens negative for any of the test targets, 99 specimens positive for at least one test target and 11 contrived mixed infections [26]. The good agreement in the presence of higher numbers of negative specimens indicates that the performance of the Xpert^®^ Xpress CoV-2/Flu/RSV *plus* is acceptable and was skewed in the current study by the low number of negatives tested. The decreased NPA observed in this study is linked to five specimens with high Ct SARS-CoV-2-positive specimens by Xpert^®^ Xpress CoV-2/Flu/RSV *plus* that were negative by SOC. A limitation of this study was the low specimen volume available, meaning that repeat testing was not possible, nor was the clinical profile available. While unlikely, given that each specimen was prepared and tested individually, cross-contamination cannot be ruled out. It is, however, possible that these specimens had low SARS-CoV-2 nucleic acid levels near the limit of the tests [44,45] and could reflect early- or late-stage infection. This could not be assessed as clinical data was not available for the specimens used. Precision in the low viral burden range (100–1000 cp/mL) was acceptable, with %CV less than 2% for CoV-2, Flu A, Flu B and RSV. The second Flu A (Flu A2) target showed greater variability in the low AccuPlex ranges, with an increased %CV (4.5%), but overall Flu A results were not affected as positivity is reported based on either target. A comprehensive analysis of the original Xpert^®^ Xpress SARS-CoV-2/Flu/RSV test recorded comparable performance between this and the Xpert^®^ Xpress SARS-CoV-2 test for the detection of SARS-CoV-2 [27]. The Xpert^®^ Xpress CoV-2/Flu/RSV *plus* test produces results in 25–45 min (as expected), which is more rapid than the existing Xpert^®^ Xpress SARS-CoV-2/Flu/RSV test (45–50 min) [28], with reports of result availability in as soon as 36 min [31]. As SARS-CoV-2 prevalence wanes and other respiratory diseases re-emerge, the use of multiplex testing off a single specimen is key to rapid results and pathogen-based treatment.

It is concerning that the NPA of both tests under evaluation was lower than that observed in other studies. However, this is likely linked to study design, with a focus on SARS-CoV-2-positive specimens that negatively influenced the NPA of the tests, to the use of residual patient specimens only, and to the variability of molecular tests near the LOD [44,45]. Regarding the performance of the tests across VOC, neither test showed decreased performance across any of the VOC assessed. This was also noted by Sluimer et al. [28] in their assessment of the Xpert^®^ Xpress SARS-CoV-2/Flu/RSV test, where a limited number of VOC were included [28], by Johnson et al. [26] in their assessment of the Xpert^®^ Xpress CoV-2/Flu/RSV *plus* test, and by Burns et al. [25] for both the Xpert^®^ Xpress SARS-CoV-2 *plus* and the Xpert^®^ Xpress CoV-2/Flu/RSV *plus*, with a focus on NGTF and VOC [25].

## 5. Conclusions

The Xpert^®^ Xpress CoV-2 *plus* and Xpert^®^ Xpress CoV-2/Flu/RSV *plus* tests can be considered to replace existing Xpert^®^ Xpress tests or other SOC tests in use. However, it is recommended that laboratories perform their own evaluations ahead of implementation and monitor test performance at scale, to ensure comparable performance compared to existing SOC and ongoing quality patient management.

## Figures and Tables

**Table 1:** Accuracy of the Xpert^®^ Xpress CoV-2 *plus* test compared to qualitative reference results. Positive percentage agreement (PPA), negative percentage agreement (NPA) and agreement are shown, including 95% CI. Clinically relevant specimens refer to both residual clinical specimens and reference materials. SOC: standard of care.

Type	n	Reference	PPA (95% CI) NPA (95% CI)	Cohen Kappa [38] (95% CI)	Agreement Score [39]
Clinically relevant specimens	157	SOC/expected results	96.4% (91.7, 98.8) 95.0% (75.1, 99.9)	0.8416 (0.7185, 0.9647)	Very good
Residual clinical specimens	125	SOC	95.2% (89.2, 98.4) 95.0 % (75.1, 99.9)	0.8348 (0.7070, 0.9626)	Very good
Clinically relevant specimens	55	Xpert^®^ Xpress SARS-CoV-2	97.9% (88.9, 99.9) 85.7% (42.1, 99.6)	0.8363 (0.6153, 1.0573)	Very good

**Table 2:** Summary of discordant specimen results by Xpert^®^ Xpress SARS-CoV-2, Xpert^®^ Xpress CoV-2 *plus* and Xpert^®^ Xpress CoV-2/Flu/RSV *plus* tests compared to standard of care (SOC) or reference results.

**SARS-CoV-2 Discordant Specimens**													
**Specimen**	**Wave**	**SOC (cobas^®^ SARS-CoV-2)**			**Xpert^®^ Xpress SARS-CoV-2**				**Xpert^®^ Xpress CoV-2 *plus***				
		**Ct E**	**Ct ORF1ab**	**Result**	**Ct E**	**Ct N2**	**Ct SPC**	**Result**	**Ct E**	**Ct N2**	**Ct RdRp**	**Ct SPC**	**Result**
52	i2	0.0	0.0	SARS-CoV-2 Negative	36.2	37.7	27.6	SARS-CoV-2 Positive	34.0	37.0	38.0	28.9	SARS-CoV-2 Positive
110	4	37.6	33.8	SARS-CoV-2 Positive	0.0	0.0	30.5	SARS-CoV-2 Negative	0.0	0.0	0.0	28.8	SARS-CoV-2 Negative
114	2	36.0	34.1	SARS-CoV-2 Positive	0.0	0.0	27.5	SARS-CoV-2 Negative	37.5	42.3	0.0	28.6	SARS-CoV-2 Positive
116	4	35.7	35.1	SARS-CoV-2 Positive	0.0	0.0	27.5	SARS-CoV-2 Negative	0.0	0.0	0.0	28.5	SARS-CoV-2 Negative
119	3	37.2	35.1	SARS-CoV-2 Positive	38.4	41.4	27.9	SARS-CoV-2 Positive	0.0	0.0	0.0	28.4	SARS-CoV-2 Negative
122	2	36.4	35.4	SARS-CoV-2 Positive	not done				0.0	0.0	0.0	28.2	SARS-CoV-2 Negative
125	2	36.9	35.6	SARS-CoV-2 Positive	not done				0.0	0.0	0.0	29.9	SARS-CoV-2 Negative
**Respiratory Discordant Specimens**													
**Specimen**	**Wave**	**SOC (cobas^®^ SARS-CoV-2)**			**Xpert^®^ Xpress CoV2/Flu/RSV *plus***								
		**E-gene**	**ORF1a**	**Result**	**CoV2**	**Flu A1**	**Flu A2**	**Flu B**	**RSV**	**SPC**	**Result**		
82	i2	0.0	0.0	SARS-CoV-2 Negative	38.5	0	0	0	0	33.2	SARS-CoV-2 Positive	FluA, FluB, RSV Negative	
83	i2	0.0	0.0	SARS-CoV-2 Negative	39.8	0	0	0	0	30.5	SARS-CoV-2 Positive	FluA, FluB, RSV Negative	
**Specimen**	**Allplex SARS-CoV-2/Flu/RSV**		**Result**	**CoV2**	**Flu A1**	**Flu A2**	**Flu B**	**RSV**	**SPC**	**Result**			
27	32.76		RSVB	36.1	0	0	0	33.2	28.9	SARS-CoV-2, RSV Positive		FluA, FluB Negative	
30	18.79		RSVA	40.9	0	0	0	24.3	29.0	SARS-CoV-2, RSV Positive		FluA, FluB Negative	
34	24.62		RSVB	43.5	0	0	0	25.8	28.6	SARS-CoV-2, RSV Positive		FluA, FluB Negative	
AccuPlex	100 cp/mL	Expected SARS-CoV-2, FluA, FluB, RSV positive		38.7	37.5	0	35.8	38	28.9	SARS-CoV-2, FluA, FluB Positive		RSV Negative	
AccuPlex	100 cp/mL			38.2	38.4	38.7	35.4	39.7	29.5	SARS-CoV-2, FluA, FluB Positive		FluA2 Negative	
AccuPlex	250 cp/mL			37.2	37.3	40.6	34.4	36.8	28.9	SARS-CoV-2, FluA, FluB Positive		FluA2 Negative	

SOC (cobas^®^ SARS-CoV-2), Xpert^®^ Xpress SARS-CoV-2 and Xpert^®^ Xpress CoV-2 *plus* results are shown, including Ct values. AccuPlex refers to the AccuPlex SARS-CoV-2, FluA, FluB, RSV positive reference material. SARS-CoV-2/CoV-2: severe acute respiratory syndrome coronavirus 2; cp/mL: copies per millilitre; Ct: cycle threshold; Flu A: influenza A; Flu B: influenza B; i2: inter-wave 2 (between waves 2 and 3); RSV: respiratory syncytial virus; SOC: standard of care, SPC: sample processing control. Gene targets refer to the SARS-CoV-2 envelope (E), nucleocapsid 2 (N2), open reading frame 1ab (ORF1ab) and RNA-dependant RNA polymerase (RdRp) genes.

**Table 3:** Precision of the Xpert^®^ Xpress SARS-CoV-2 and Xpert^®^ Xpress CoV-2 *plus* tests.

AccuPlex Dilution (Wildtype)		E-Gene			N2-Gene			RdRp			SPC (Internal Control)		
		Mean	SD	%CV	Mean	SD	%CV	Mean	SD	%CV	Mean	SD	%CV
Xpert^®^ Xpress SARS-CoV-2 test	100 cp/mL	37.97	3.50	9.22	39.00	0.56	1.43	Target not included			27.93	0.25	0.90
	250 cp/mL	35.53	0.23	0.65	38.15	Not calculated					28.13	0.93	3.30
	500 cp/mL	34.47	0.46	1.34	37.27	0.23	0.62				27.87	0.25	0.90
	1000 cp/mL	33.23	0.40	1.22	35.80	0.40	1.12				27.90	0.26	0.95
	Overall	35.30	1.15	3.11	37.55	0.40	1.05				27.96	0.42	1.51
Xpert^®^ Xpress CoV-2 *plus* test	100 cp/mL	34.63	0.15	0.44	39.37	0.91	2.30	38.17	0.15	0.40	28.43	0.29	1.02
	250 cp/mL	33.97	0.32	0.95	37.23	0.15	0.41	37.03	0.40	1.09	28.90	0.30	1.04
	1000 cp/mL	31.60	0.17	0.55	35.17	0.50	1.43	34.67	0.46	1.33	28.50	0.36	1.27
	1000 cp/mL	31.60	0.17	0.55	35.17	0.50	1.43	34.67	0.46	1.33	28.50	0.36	1.27
	Overall	33.29	0.20	0.60	37.06	0.49	1.31	36.50	0.37	1.03	28.61	0.26	0.92

Mean Ct values, standard deviation (SD) and percentage coefficient of variation (%CV) per gene target and dilution are shown for the Xpert^®^ Xpress SARS-CoV-2 and Xpert^®^ Xpress CoV-2 *plus* tests. Overall mean Ct, SD and %CV are also provided. Gene targets refer to the SARS-CoV-2 envelope (E), nucleocapsid 2 (N2), open reading frame 1ab (ORF1ab) and RNA-dependant RNA polymerase (RdRp) genes. Ct: cycle threshold; cp/mL: copies per millilitre.

**Table 4:** Accuracy of the Xpert^®^ Xpress CoV-2/Flu/RSV plus test compared to qualitative reference results. Positive agreement (PPA), negative agreement (NPA) and agreement are shown, including 95 percent confidence intervals (95% CI). Clinically relevant specimens refer to both residual clinical specimens and reference materials. AccuPlex refers to the AccuPlex SARS-CoV-2, Flu A, Flu B, RSV positive reference material.

Type	n	Reference	PPA (95% CI) NPA (95% CI)	Cohen Kappa [38] (95% CI)	Agreement Score [39]
**Combined results any pathogen**					
Residual clinical specimens	92	SOC	100% (95.6, 100) 83.3% (51.6, 97.9)	0.8972 (0.7569, 1.0374)	Very good
Residual clinical specimens	95 ^1^		100% (95.5, 100) 66.7% (38.4, 88.2)	0.7711 (0.5810, 0.9612)	Good
**Results per pathogen (CoV-2, Flu A, Flu B, RSV) and across all diseases** **Clinically relevant specimens (residual clinical specimens and AccuPlex)**					
CoV-2	113	SOC/expected reference results	100% (94.1, 100) 90.4% (79.0, 96.8)	0.9103 (0.8338, 0.9869)	Very Good
FluA	113		91.3% (72.0, 98.9) 100% (96.0, 100)	0.9436 (0.8662, 1.0210)	Very Good
FluB	113		100% (85.2, 100) 100% (96.2, 100)	1.000 (1.000, 1.000)	Very Good
RSV	113		97.0% (84.2, 99.9) 100% (95.5, 100)	0.9784 (0.9363, 1.0205)	Very Good
All pathogens	452		97.9% (93.9, 99.6) 98.4% (96.3, 99.5)	0.9588 (0.9305, 0.9871)	Very Good
**Results per pathogen (CoV-2, Flu A, Flu B, RSV) and across all pathogens** **Residual clinical specimens only**					
CoV-2	92	SOC	100% (91.2, 100) 90.4% (79.0, 96.8)	0.8910 (0.7986, 0.9834)	Very Good
FluA	92		100% (69.2, 100) 100% (95.6, 100)	1.000 (1.000, 1.000)	Very Good
FluB	92		100% (69.2, 100) 100% (95.6, 100)	1.000 (1.000, 1.000)	Very Good
RSV	92		100% (83.2, 100) 100% (95.0, 100)	1.000 (1.000, 1.000)	Very Good
All pathogens	368		100% (95.5, 100) 98.3% (96.0, 99.4)	0.9610 (0.9270, 0.9949)	Very Good

^1^ Three specimens were RSV positive, SARS-CoV-2 positive by SOC, but RSV/SARS-CoV-2 co-infected by Xpert^®^ Xpress CoV-2/Flu/RSV plus and these two targets were thus also assessed as individual targets. CoV-2 (SARS-CoV-2): severe acute respiratory syndrome coronavirus 2; cp/mL: copies per millilitre; Ct: cycle threshold; Flu A: influenza A; Flu B: influenza B; RSV: respiratory syncytial virus; SOC: standard of care.

**Table 5:** Precision of the Xpert^®^ Xpress CoV-2/Flu/RSV plus test.

AccuPlex Respiratory	CoV-2			Flu A1			Flu A2			Flu B			RSV		
	Mean	SD	%CV	Mean	SD	%CV	Mean	SD	%CV	Mean	SD	%CV	Mean	SD	%CV
100 cp/mL	38.30	0.36	0.94	37.87	0.47	1.25	38.65	Not determined		35.43	0.35	0.99	38.63	0.93	2.41
250 cp/mL	37.13	0.06	0.16	36.93	0.72	1.96	38.63	1.74	4.50	34.70	0.36	1.04	36.97	0.15	0.41
500 cp/mL	36.03	0.31	0.85	35.80	0.17	0.48	36.70	0.17	0.47	33.50	0.10	0.30	35.77	0.12	0.32
1000 cp/mL	35.20	0.10	0.28	32.83	0.10	0.30	35.63	0.06	0.16	32.83	0.71	2.16	35.10	0.66	1.87
Overall	37.72	0.21	0.55	37.40	0.60	1.60	38.64	1.74	4.50	35.07	0.36	1.02	37.80	0.54	1.41

Mean Ct values, standard deviation (SD) and percentage coefficient of variation (%CV) are shown across disease targets. Overall mean Ct, SD and %CV are also provided. CoV-2: SARS-CoV-2; Ct: cycle threshold; cp/mL: copies per millilitre; Flu A1/2: influenza A (targets 1 or 2); Flu B: influenza B; RSV: respiratory syncytial virus.

## Data Availability

Complete data is available in the Supplementary Tables.

## Supplementary Materials

The following supporting information can be downloaded at: https://www.mdpi.com/article/10.3390/diagnostics13010034/s1. Table S1: Performance of the Xpert^®^ Xpress CoV-2 *plus* test compared to standard of care (SOC) results and reference material. Table S2: Performance of the Xpert^®^ Xpress CoV-2/Flu/RSV *plus* test compared to standard of care (SOC) results and reference material.
