# Supplementary material for: Rapid Evaluation of the Xpert® Xpress CoV-2 plus and Xpert® Xpress CoV-2/Flu/RSV plus Tests
Source: Diagnostics (Basel). 2022 Dec 22;13(1):34. doi: 10.3390/diagnostics13010034 (PMC9818900; doi:10.3390/diagnostics13010034)
Supplement: Supplementary file 1 [file diagnostics-13-00034-s001.zip › diagnostics-2104334-supplementary.pdf]

Supplementary Table S1

Supplementary Table S1: Performance of the Xpert® Xpress CoV-2 plus test compared to standard of care (SOC) results and reference material.

SOC (cobas® SARS-CoV-2, TaqPath COVID-19), Xpert® Xpress SARS-CoV-2 and Xpert® Xpress CoV-2 plus results are shown, including Ct values. Residual clinical specimens are sorted according to SOC ORF1ab Ct values. SARS-CoV-2 positive results are shown in red and SARS-CoV-2 negative results are shown in green.

Specimens include residual respiratory clinical specimens and AccuPlex SARS-CoV-2 reference material. SARS-CoV-2 variants of concern (VOC) include genotyped wildtype (Wuhan with D614G), Alpha, Beta, Gamma (AccuPlex only), Delta and Omicron BA.1 and BA.4 (clinical only) VOC. Where specimens were not genotyped, the wave across the four major waves in South Africa is given as an indicator of likely VOC: Wildtype (Wuhan with D614G): April-July 2020; Beta: November 2020-January 2021; Alpha/Delta: May-July 2021; Omicron: October 2021-April 2022. Limited reference material (AccuPlex SARS-CoV-2 or AccuPlex SARS-CoV-2 Variants Panels 1 and 2, LGC SeraCare, Milford, MA, USA) was also included in the study.

Gene targets refer to the SARS-CoV-2 envelope (E), nucleocapsid 2 (N2), open reading frame 1ab (ORF1ab) and RNA-dependant RNA polymerase (RdRp) genes. Ct: cycle threshold; i2: interwave period 2 (the time between waves 2 and 3); SOC: standard of care, SPC: sample processing control; VOC: variants of concern.

SARS-CoV-2 Specimens (typed)														
Specimen Information			SOC Result				Xpert® Xpress SARS-CoV-2		SOC Assay	Xpert® Xpress CoV-2 *plus*				
Specimen	Wave	Variant	cobas Ct E	SOC Ct ORF1ab	TaqPath Ct N	TaqPath Ct S	Ct E	Ct N2	Result	Ct E	Ct N2	Ct RdRp	Ct SPC	Result
1	3	Delta		14.3	17.7	18.6			Positive	24.9	29.5	27.9	28.4	Positive
2	4	Omicron BA.1		16.3	14.7	0.0	14.9	16.6	Positive	16.2	19.8	19.3	31.4	Positive
3	3	Delta		17.0	16.7	20.3			Positive	18.1	21.8	22.5	36.6	Positive
4	2	Beta	17.6	17.7					Positive	16.5	19.9	19.1	31.6	Positive
5	2	Beta		18.0	24.6	16.1			Positive	28.5	32.0	31.6	28.4	Positive
6	3	Alpha		19.3	18.8	0.0			Positive	17.6	20.4	23.5	31.8	Positive
7	1	Wildtype D614G	20.0	19.6					Positive	16.3	19.6	19.1	43.8	Positive
8	1	Wildtype D614G	20.5	20.1					Positive	20.6	23.6	23.4	28.7	Positive
9	3	Delta		20.4	20.0	16.9			Positive	23.4	27.2	26.5	28.9	Positive
10	3	Alpha		20.8	19.9	0.0			Positive	18.8	21.7	22.3	31.4	Positive
11	4	Omicron BA.1		21.3	21.4	0.0	27.1	28.6	Positive	25.8	30.6	30.2	28.2	Positive
12	1	Wildtype D614G	21.9	21.5					Positive	23.2	26.6	26.3	28.5	Positive
13	3	Alpha		21.8	24.0	0.0			Positive	20.6	24.5	24.8	31.1	Positive
14	2	Beta		22.3	22.3	22.3			Positive	22.1	25.1	25.9	29.9	Positive
15	3	Delta		22.7	22.3	22.7			Positive	22.0	25.1	26.1	29.4	Positive
16	4	Omicron BA.1		23.1	23.4	0.0	22.7	24.7	Positive	23.2	26.5	26.4	29.1	Positive
17	3	Alpha		23.1	23.0	0.0			Positive	21.3	24.6	24.5	29.2	Positive
18	1	Wildtype D614G	23.9	23.3					Positive	23.5	26.9	26.3	28.5	Positive
19	3	Alpha		23.6	23.4	0.0			Positive	24.4	28.1	28.2	29.6	Positive
20	3	Delta		23.6	24.3	22.6			Positive	24.9	28.6	28.4	29.8	Positive
21	2	Beta		23.9	24.4	23.6			Positive	24.9	28.1	27.9	28.4	Positive
22	2	Beta		24.0	25.0	25.8			Positive	26.1	29.4	28.8	28.3	Positive
23	1	Wildtype D614G	24.8	24.7					Positive	24.9	28.3	28.2	28.5	Positive
24	3	Delta	26.0	25.0					Positive	24.4	28.0	27.4	28.7	Positive
25	1	Wildtype D614G	26.1	25.4					Positive	23.2	26.2	26.0	29.6	Positive
26	3	Delta		25.5	24.7	26.0			Positive	25.6	31.6	29.5	29.6	Positive
27	3	Alpha		25.5	26.3	0.0			Positive	26.6	29.6	29.4	29.6	Positive
28	3	Alpha		25.5	25.5	0.0			Positive	25.7	29.7	30.8	29.7	Positive
29	3	Delta	27.0	26.0					Positive	29.1	32.8	32.8	28.8	Positive
30	1	Wildtype D614G	27.0	26.6					Positive	30.1	33.4	33.1	28.3	Positive
31	2	Beta		26.8	25.9	27.2			Positive	26.9	30.1	29.9	28.5	Positive
32	2	Beta		27.2	26.7	26.6			Positive	27.8	31.1	31.1	28.4	Positive
33	4	Omicron BA.1		27.3	26.2	0.0	23.2	25.1	Positive	24.5	29.8	29.3	28.9	Positive
34	1	Wildtype D614G	28.2	27.5					Positive	36.1	40.0	40.8	28.8	Positive
35	3	Alpha		27.7	26.6	0.0			Positive	27.3	31.5	31.4	29.4	Positive
36	3	Delta	29.0	28.0					Positive	27.6	32.6	30.2	29.0	Positive
37	2	Beta		28.1	26.8	27.1			Positive	29.0	32.4	31.6	28.8	Positive
38	2	Beta		28.7	28.5	29.3			Positive	28.5	31.4	31.3	28.7	Positive
39	1	Wildtype D614G	29.9	29.2					Positive	35.3	38.9	38.1	28.6	Positive
40	1	Wildtype D614G	29.8	29.4					Positive	27.8	31.1	30.9	28.8	Positive
41	4	Omicron BA.4	20.0	20.4			21.4	23.5	Positive	19.1	22.5	21.9	32.2	Positive
42	4	Omicron BA.4	20.7	20.9			21.7	23.6	Positive	21.6	25.1	25.6	28.9	Positive
43	4	Omicron BA.4	20.6	20.9			22.4	24.5	Positive	20.2	23.7	23.8	29.3	Positive
44	4	Omicron BA.4	19.0	19.0			17.9	19.7	Positive	16.8	20.2	20.6	39.0	Positive
45	4	Omicron BA.4	19.4	19.7			22.1	23.8	Positive	19.3	22.4	23.0	30.2	Positive
46	2	Beta		30.0	29.3	29.8			Positive	30.1	33.2	33.1	28.6	Positive
47	3	Delta	31.0	30.0					Positive	33.2	36.2	35.9	28.9	Positive
48	4	Omicron BA.1		31.7	30.7	0.0	27.4	28.9	Positive	27.5	31.1	30.8	29.6	Positive
49	3	Alpha		32.0	29.4	0.0			Positive	28.5	31.5	32.8	29.1	Positive
50	3	Alpha		32.0	32.1	0.0			Positive	33.8	36.6	37.9	29.9	Positive

SARS-CoV-2 Specimens (across wave)														
Specimen Information			Reference			Xpert® Xpress SARS-CoV-2				Xpert® Xpress CoV2-plus				
Specimen	Wave	Result	cobas Ct E	SOC Ct ORF1ab	TaqPath Ct N	Ct E	Ct N2	Ct SPC	Result	Ct E	Ct N2	Ct RdRp	Ct SPC	Result
51	i2	Negative	0.0	0.0		0.0	0.0	29.3	Negative	0.0	0.0	0.0	29.8	Negative
52	i2	Negative	0.0	0.0		36.2	37.7	27.6	Positive	34.0	37.0	38.0	28.9	Positive
53	i2	Negative	0.0	0.0		0.0	0.0	27.1	Negative	0.0	0.0	0.0	29.1	Negative
54	i2	Negative	0.0	0.0		0.0	0.0	28.0	Negative	0.0	0.0	0.0	29.0	Negative
55	i2	Negative	0.0	0.0		0.0	0.0	30.1	Negative	0.0	0.0	0.0	28.7	Negative
56	i2	Negative	0.0	0.0					Negative	0.0	0.0	0.0	30.2	Negative
57	i2	Negative	0.0	0.0					Negative	0.0	0.0	0.0	29.9	Negative
58	i2	Negative	0.0	0.0					Negative	0.0	0.0	0.0	29.2	Negative
59	i2	Negative	0.0	0.0					Negative	0.0	0.0	0.0	29.1	Negative
60	i2	Negative	0.0	0.0					Negative	0.0	0.0	0.0	29.2	Negative
61	i2	Negative	0.0	0.0					Negative	0.0	0.0	0.0	29.6	Negative
62	i2	Negative	0.0	0.0					Negative	0.0	0.0	0.0	29.4	Negative
63	i2	Negative	0.0	0.0					Negative	0.0	0.0	0.0	28.9	Negative
64	i2	Negative	0.0	0.0					Negative	0.0	0.0	0.0	29.3	Negative
65	i2	Negative	0.0	0.0					Negative	0.0	0.0	0.0	29.9	Negative
66	i2	Negative	0.0	0.0					Negative	0.0	0.0	0.0	29.1	Negative
67	i2	Negative	0.0	0.0					Negative	0.0	0.0	0.0	29.4	Negative
68	i2	Negative	0.0	0.0					Negative	0.0	0.0	0.0	29.2	Negative
69	i2	Negative	0.0	0.0					Negative	0.0	0.0	0.0	29.2	Negative
70	i2	Negative	0.0	0.0					Negative	0.0	0.0	0.0	29.3	Negative
71	3	Positive		14.5	15.3				Positive	15.9	18.6	21.0	33.4	Positive
72	3	Positive		14.6	16.7	14.7	17.4	0.0	Positive	15.7	20.1	19.2	34.3	Positive
73	4	Positive	16.0	16.1					Positive	17.2	20.4	20.1	0.0	Positive
74	3	Positive		16.5	18.2				Positive	12.5	16.2	15.3	30.8	Positive
75	1	Positive	16.9	16.8		17.5	20.5	0.0	Positive	15.2	20.6	19.5	32.0	Positive
76	3	Positive		17.3	19.4				Positive	16.0	21.6	18.6	31.2	Positive
77	1	Positive	18.2	17.8					Positive	11.8	13.3	15.9	41.8	Positive
78	1	Positive	18.2	17.8					Positive	14.0	17.4	16.4	44.4	Positive
79	2	Positive	18.7	18.0		19.8	21.8	0.0	Positive	18.5	22.0	21.1	31.2	Positive
80	2	Positive	18.4	18.4					Positive	17.2	21.1	21.1	30.1	Positive
81	4	Positive	18.7	18.4					Positive	17.9	21.5	21.1	30.6	Positive
82	4	Positive	18.3	18.6					Positive	16.2	19.7	19.3	31.1	Positive
83	1	Positive	19.1	18.7					Positive	16.5	19.8	19.2	44.0	Positive
84	2	Positive	18.9	19.3		20.3	20.8	0.0	Positive	19.4	22.0	24.1	30.3	Positive
85	4	Positive	20.3	19.8		21.5	21.8	27.9	Positive	19.8	21.7	22.5	29.9	Positive
86	2	Positive	22.1	21.4					Positive	26.2	29.3	29.0	35.2	Positive
87	1	Positive	22.0	21.8					Positive	17.7	20.9	20.4	34.9	Positive
88	4	Positive	23.0	22.5		21.0	23.9	27.5	Positive	20.6	25.2	24.1	28.7	Positive
89	3	Positive	23.1	22.8					Positive	18.8	22.3	22.0	29.1	Positive
90	2	Positive	23.0	23.1		21.2	22.5	27.7	Positive	21.2	24.4	25.5	29.8	Positive
91	1	Positive	25.0	24.0		29.2	29.9	29.2	Positive	25.6	27.9	28.8	29.3	Positive
92	4	Positive	25.1	24.6					Positive	26.3	30.1	29.2	28.1	Positive
93	3	Positive	25.0	24.9					Positive	17.9	21.4	21.0	0.0	Positive
94	2	Positive	20.7	25.1					Positive	23.7	27.4	26.4	28.4	Positive
95	2	Positive	27.0	26.1					Positive	27.0	31.2	30.4	29.1	Positive
96	1	Positive	27.0	26.4					Positive	29.5	32.7	32.3	28.2	Positive
97	3	Positive	27.0	26.6		24.3	25.8	27.2	Positive	22.6	25.7	25.6	29.2	Positive
98	2	Positive	29.0	28.4					Positive	27.4	31.4	30.6	29.6	Positive
99	3	Positive	29.1	28.4					Positive	26.3	30.1	29.2	28.1	Positive
100	4	Positive	29.1	28.4		33.2	36.6	28.2	Positive	32.0	35.4	34.5	28.9	Positive
101	1	Positive	31.0	30.0					Positive	29.4	33.1	32.6	28.7	Positive
102	3	Positive	31.0	30.0					Positive	31.5	35.0	34.8	28.4	Positive
103	2	Positive	31.0	30.1		0.0	39.4	27.7	Positive	36.4	37.7	37.3	28.0	Positive
104	4	Positive	31.1	30.3					Positive	30.5	34.7	34.2	28.3	Positive
105	4	Positive	33.3	31.0					Positive	36.3	42.7	39.8	28.3	Positive
106	1	Positive	33.0	31.9		39.4	36.0	27.3	Positive	34.3	37.0	39.1	28.2	Positive
107	4	Positive	36.0	32.5					Positive	37.6	0.0	0.0	29.8	Positive
108	3	Positive	35.0	33.0					Positive	39.7	0.0	0.0	28.2	Positive
109	4	Positive	35.0	33.0					Positive	35.0	38.6	38.1	28.0	Positive
110	4	Positive	37.6	33.8		0.0	0.0	30.5	Negative	0.0	0.0	0.0	28.8	Negative
111	2	Positive	35.0	33.8					Positive	36.8	39.4	40.8	29.0	Positive
112	1	Positive	36.0	33.9					Positive	38.4	42.0	39.3	28.1	Positive
113	3	Positive	36.0	34.0		39.9	41.2	27.2	Positive	0.0	0.0	42.3	28.1	Positive
114	2	Positive	36.0	34.1		0.0	0.0	27.5	Negative	37.5	42.3	0.0	28.6	Positive
115	1	Positive	35.0	34.2					Positive	34.4	37.6	38.2	28.2	Positive
116	4	Positive	35.7	35.1		0.0	0.0	27.5	Negative	0.0	0.0	0.0	28.5	Negative
117	2	Positive	36.3	35.1		0.0	40.1	28.7	Positive	36.0	40.2	41.1	29.1	Positive
118	2	Positive	36.4	35.1					Positive	38.6	41.6	39.1	28.6	Positive
119	3	Positive	37.2	35.1		38.4	41.4	27.9	Positive	0.0	0.0	0.0	28.4	Negative
120	1	Positive	38.3	35.1		0.0	39.4	27.4	Positive	38.5	0.0	0.0	28.3	Positive
121	1	Positive	36.9	35.2					Positive	38.4	0.0	0.0	29.0	Positive
122	2	Positive	36.4	35.4					Positive	0.0	0.0	0.0	28.2	Negative
123	4	Positive	38.3	35.6		38.6	40.3	27.3	Positive	36.8	40.2	41.5	28.5	Positive
124	1	Positive	36.6	35.6					Positive	37.6	0.0	0.0	28.5	Positive
125	2	Positive	36.9	35.6					Positive	0.0	0.0	0.0	29.9	Negative

Accuplex SARS-CoV-2 Reference Material (5000cp/mL and diluted with saline)													
Accuplex SARS-CoV-2			Xpert® Xpress SARS-CoV-2					Xpert® Xpress CoV-2 *plus*					
Concentration	Variant	6/10 colour	Ct E	Ct N2	Ct SPC	Result	6/10 colour	Ct E	Ct N2	Ct RdRp	Ct SPC	Result	
100cp/mL	Wuhan	6	42.0	38.5	27.9	Positive	10	34.5	39.5	38.0	28.6	Positive	
100cp/mL	Wuhan	10	36.2	39.6	28.2	Positive	10	34.6	38.4	38.2	28.6	Positive	
100cp/mL	Wuhan	6	35.7	38.9	27.7	Positive	6	34.8	40.2	38.3	28.1	Positive	
250cp/mL	Wuhan	10	35.4	38.3	27.5	Positive	6	33.6	37.1	36.8	28.6	Positive	
250cp/mL	Wuhan	10	35.4	0.0	29.2	Positive	10	34.2	37.2	37.5	29.2	Positive	
250cp/mL	Wuhan	6	35.8	38.0	27.7	Positive	6	34.1	37.4	36.8	28.9	Positive	
500cp/mL	Wuhan	10	34.2	37.4	27.9	Positive	10	33.1	36.9	36.4	28.6	Positive	
500cp/mL	Wuhan	10	35.0	37.4	28.1	Positive	6	33.0	36.1	35.6	28.7	Positive	
500cp/mL	Wuhan	6	34.2	37.0	27.6	Positive	6	32.8	36.4	36.4	28.5	Positive	
1000cp/mL	Wuhan	6	32.8	35.4	27.6	Positive	10	31.7	34.7	35.2	28.9	Positive	
1000cp/mL	Wuhan	10	33.3	35.8	28.1	Positive	10	31.4	35.1	34.4	28.2	Positive	
1000cp/mL	Wuhan	6	33.6	36.2	28.0	Positive	6	31.7	35.7	34.4	28.4	Positive	
500cp/mL	Alpha	6	34.5	36.8	28.1	Positive	6	32.6	35.8	36.1	28.3	Positive	
500cp/mL	Alpha	10	34.1	36.4	27.2	Positive	10	33.2	36.5	36.3	28.6	Positive	
500cp/mL	Beta	6	33.1	36.8	27.5	Positive	6	32.4	35.7	35.6	28.5	Positive	
500cp/mL	Beta	10	33.8	36.1	27.3	Positive	10	32.3	36.7	35.8	28.7	Positive	
500cp/mL	Gamma	6	33.8	36.4	27.2	Positive	6	33.4	35.7	35.9	29.0	Positive	
500cp/mL	Gamma	10	34.2	37.6	28.2	Positive	10	32.5	35.9	39.1	28.4	Positive	
500cp/mL	Delta	6	32.9	36.7	27.2	Positive	6	32.7	36.3	35.7	28.3	Positive	
500cp/mL	Delta	10	33.8	37.0	27.5	Positive	10	32.2	35.5	34.9	28.2	Positive	

SARS-CoV-2 Live Viral Culture swabs (eluted in water)												
Accuplex SARS-CoV-2		Xpert® Xpress SARS-CoV-2					Xpert® Xpress CoV-2 *plus*					
Concentration	Variant	6/10 colour	Ct E	Ct N2	Ct SPC	Result	6/10 colour	Ct E	Ct N2	Ct RdRp	Ct SPC	Result
log 2.5cp/ml	Wuhan	6	0.0	0.0	27.9	Positive	6	35.5	39.5	38.9	28.5	Positive
log 2.5cp/ml	Wuhan	10	0.0	0.0	29.8	Positive	10	36.5	39.9	40.0	29.1	Positive
log 2.5cp/ml	Wuhan	10	0.0	0.0	28.3	Positive	10	36.0	39.3	44.5	28.6	Positive
log 3.4cp/ml	Wuhan	6	40.1	43.5	29.2	Positive	6	34.5	38.7	38.3	28.7	Positive
log 3.4cp/ml	Wuhan	6	37.3	41.1	27.7	Positive	6	34.2	37.7	38.4	28.7	Positive
log 3.4cp/ml	Wuhan	10	0.0	41.7	27.7	Positive	10	35.0	39.0	37.3	28.9	Positive
log 4.4cp/ml	Wuhan	6	31.4	34.6	27.6	Positive	6	30.6	34.7	34.2	29.1	Positive
log 4.4cp/ml	Wuhan	10	32.3	34.9	29.4	Positive	10	30.2	34.1	33.7	29.1	Positive
log 4.4cp/ml	Wuhan	10	33.1	35.3	27.6	Positive	10	30.0	33.8	33.9	29.3	Positive
log 5.0cp/ml	Wuhan	6	32.5	34.2	27.8	Positive	6	26.4	30.3	30.0	28.8	Positive
log 5.0cp/ml	Wuhan	6	30.4	32.5	28.5	Positive	6	26.2	30.0	29.9	29.2	Positive
log 5.0cp/ml	Wuhan	10	29.6	32.1	27.5	Positive	10	26.2	30.3	29.9	28.9	Positive

Supplementary Table S2

Supplementary Table S2: Performance of the Xpert® Xpress CoV-2/Flu/RSV plus test compared to standard of care (SOC) results and reference material.

SOC (TaqMan™ TaqPath COVID-19; AllPlex™ SARS-CoV-2; Xpert® Xpress SARS-CoV-2 and cobas® SARS-CoV-2; AllPlex™ SARS-CoV-2/FluA/FluB/RSV) and Xpert® Xpress CoV-2/Flu/RSV plus results are shown, including Ct values. Residual respiratory specimens are sorted by pathogen (Flu A, Flu B, RSV or SARS-COV-2) and Ct value. Residual respiratory clinical SARS-CoV-2 specimens are sorted according to SOC ORF1ab Ct values. Specimens include residual clinical specimens and AccuPlex SARS-CoV-2 reference material. SARS-CoV-2 variants of concern (VOC) include genotyped wildtype (Wuhan (AccuPlex only); Wuhan with D614G (respiratory specimens)), Alpha, Beta, Gamma (AccuPlex only), Delta and Omicron BA.1 VOC. Respiratory pathogen positive results are shown in red and negative results are shown in green.

Gene targets refer to the SARS-CoV-2 envelope (E), nucleocapsid 2 (N2), open reading frame 1ab (ORF1ab) and RNA-dependant RNA polymerase (RdRp), and spike (S) genes. CoV-2 (SARS-CoV-2: severe acute respiratory syndrome 2; Ct: cycle threshold; cp/mL: copies per millilitre; Flu A1/2: influenza A (targets 1 or 2); Flu B: influenza B; RSV: respiratory syncytial virus; SOC: standard of care, SPC: sample processing control; VOC: variants of concern.

Flu A, Flu B and RSV Specimens										
SOC				Xpert COV2/FLUA/FluB/RSV Testing Data						
Specimen	Virus	Ct	Subtype	Ct COV2	Ct FluA1	Ct FluA2	Ct FluB	Ct RSV	Ct SPC	Result
1	Flu A	22.3	H1N1pdm09	0.0	22.4	25.1	0.0	0.0	29.1	FluA positive
2	Flu A	22.6	H3N2	0.0	19.5	22.2	0.0	0.0	30.4	FluA positive
3	Flu A	22.9	H1N1pdm09	0.0	19.4	22.1	0.0	0.0	30.2	FluA positive
4	Flu A	23.8	H1N1pdm09	0.0	19.2	22.1	0.0	0.0	29.9	FluA positive
5	Flu A	25.7	H1N1pdm09	0.0	22.3	24.9	0.0	0.0	30.5	FluA positive
6	Flu A	29.3	H1N1pdm09	0.0	25.0	27.8	0.0	0.0	28.8	FluA positive
7	Flu A	30.8	H3N2	0.0	30.4	32.7	0.0	0.0	29.1	FluA positive
8	Flu A	31.6	H1N1pdm09	0.0	31.2	33.2	0.0	0.0	29.4	FluA positive
9	Flu A	32.5	H1N1pdm09	0.0	32.1	34.2	0.0	0.0	28.9	FluA positive
10	Flu A	34.8	H1N1pdm09	0.0	31.1	33.3	0.0	0.0	30.2	FluA positive
11	Flu B	18.5	Flu B-Victoria lineage	0.0	0.0	0.0	16.1	0.0	32.4	FluB positive
12	Flu B	20.9	Flu B-Victoria lineage	0.0	0.0	0.0	20.0	0.0	29.9	FluB positive
13	Flu B	21.1	Flu B-Victoria lineage	0.0	0.0	0.0	16.7	0.0	29.2	FluB positive
14	Flu B	26.0	Flu B-Victoria lineage	0.0	0.0	0.0	21.3	0.0	28.1	FluB positive
15	Flu B	28.8	Flu B-Victoria lineage	0.0	0.0	0.0	25.3	0.0	28.6	FluB positive
16	Flu B	31.5	Flu B-Victoria lineage	0.0	0.0	0.0	28.6	0.0	29.1	FluB positive
17	Flu B	32.2	Flu B-Yamagata lineage	0.0	0.0	0.0	27.7	0.0	28.9	FluB positive
18	Flu B	32.6	Flu B	0.0	0.0	0.0	34.2	0.0	28.6	FluB positive
19	Flu B	34.2	Flu B-Victoria lineage	0.0	0.0	0.0	31.3	0.0	29.2	FluB positive
20	Flu B	35.2	Flu B	0.0	0.0	0.0	35.5	0.0	30.1	FluB positive
21	RSV	18.6	RSV A	0.0	0.0	0.0	0.0	21.5	29.4	RSV positive
22	RSV	18.7	RSV A	0.0	0.0	0.0	0.0	23.4	30.7	RSV positive
23	RSV	18.8	RSV A	40.9	0.0	0.0	0.0	24.3	29.0	RSV/COV2 positive
24	RSV	20.8	RSV B	0.0	0.0	0.0	0.0	23.4	29.1	RSV positive
25	RSV	21.9	RSV A	0.0	0.0	0.0	0.0	20.2	29.1	RSV positive
26	RSV	22.0	RSV B	0.0	0.0	0.0	0.0	19.6	29.2	RSV positive
27	RSV	22.0	RSV A	0.0	0.0	0.0	0.0	28.1	28.3	RSV positive
28	RSV	23.7	RSV B	0.0	0.0	0.0	0.0	21.8	29.0	RSV positive
29	RSV	24.6	RSV B	43.5	0.0	0.0	0.0	25.8	28.6	RSV/COV2 positive
30	RSV	25.1	RSV A	0.0	0.0	0.0	0.0	23.4	28.6	RSV positive
31	RSV	25.2	RSV B	0.0	0.0	0.0	0.0	28.1	28.4	RSV positive
32	RSV	25.6	RSV A	0.0	0.0	0.0	0.0	24.3	29.2	RSV positive
33	RSV	25.9	RSV A	0.0	0.0	0.0	0.0	24.8	28.5	RSV positive
34	RSV	26.1	RSV A	0.0	0.0	0.0	0.0	24.3	28.7	RSV positive
35	RSV	26.9	RSV B	0.0	0.0	0.0	0.0	23.8	29.3	RSV positive
36	RSV	27.0	RSV B	0.0	0.0	0.0	0.0	28.1	29.2	RSV positive
37	RSV	32.8	RSV B	36.1	0.0	0.0	0.0	33.2	28.9	RSV/COV2 positive
38	RSV	34.2	RSV A	0.0	0.0	0.0	0.0	34.5	28.6	RSV positive
39	RSV	34.5	RSV B	0.0	0.0	0.0	0.0	26.1	28.1	RSV positive
40	RSV	36.0	RSV B	0.0	0.0	0.0	0.0	32.7	29.2	RSV positive

SARS-CoV-2 Specimens													
Specimen	Virus	Wave	Variant	TaqPath COVID-19			Ct COV2	Ct FluA1	Ct FluA2	Ct FluB	Ct RSV	Ct SPC	Result
				Ct ORF1ab	Ct N-gene	Ct S-gene							
42	CoV2	3	Delta	17.0	16.7	20.3	22.6	0.0	0.0	0.0	0.0	38.1	SARS-CoV-2 positive
52	CoV2	2	Beta	17.6	17.7	17.0	18.8	0.0	0.0	0.0	0.0	34.3	SARS-CoV-2 positive
51	CoV2	3	Alpha	20.8	19.9	SGTF	21.1	0.0	0.0	0.0	0.0	32.6	SARS-CoV-2 positive
43	CoV2	3	Delta	20.4	20.0	16.9	26.9	0.0	0.0	0.0	0.0	31.1	SARS-CoV-2 positive
44	CoV2	3	Delta	22.7	22.3	22.7	25.5	0.0	0.0	0.0	0.0	0.0	SARS-CoV-2 positive
47	CoV2	2	Beta	22.3	22.3	22.3	23.2	0.0	0.0	0.0	0.0	30.1	SARS-CoV-2 positive
50	CoV2	3	Alpha	21.8	24.0	SGTF	23.4	0.0	0.0	0.0	0.0	39.5	SARS-CoV-2 positive
45	CoV2	2	Beta	18.0	24.6	16.1	30.9	0.0	0.0	0.0	0.0	30.3	SARS-CoV-2 positive
46	CoV2	3	Delta	25.5	24.7	26.0	29.6	0.0	0.0	0.0	0.0	30.3	SARS-CoV-2 positive
53	CoV2	2	Beta	26.8	25.9	27.2	29.7	0.0	0.0	0.0	0.0	29.0	SARS-CoV-2 positive
49	CoV2	3	Alpha	27.7	26.6	SGTF	30.0	0.0	0.0	0.0	0.0	29.5	SARS-CoV-2 positive
41	CoV2	2	Beta	28.7	28.5	29.3	30.7	0.0	0.0	0.0	0.0	30.9	SARS-CoV-2 positive
48	CoV2	3	Alpha	32.0	29.4	SGTF	30.1	0.0	0.0	0.0	0.0	29.0	SARS-CoV-2 positive
				cobas® SARS-CoV-2									
				E-gene	ORF1a								
55	CoV2	3	Alpha	23.6	23.4		27.3	0.0	0.0	0.0	0.0	30.5	SARS-CoV-2 positive
59	CoV2	1	Wildtype D614G	24.8	24.7		26.1	0.0	0.0	0.0	0.0	28.3	SARS-CoV-2 positive
56	CoV2	1	Wildtype D614G	26.1	25.4		26.0	0.0	0.0	0.0	0.0	30.0	SARS-CoV-2 positive
58	CoV2	1	Wildtype D614G	27.0	26.6		32.4	0.0	0.0	0.0	0.0	29.3	SARS-CoV-2 positive
57	CoV2	1	Wildtype D614G	28.2	27.5		38.3	0.0	0.0	0.0	0.0	28.9	SARS-CoV-2 positive
54	CoV2	3	Delta	29.0	28.0		30.4	0.0	0.0	0.0	0.0	29.4	SARS-CoV-2 positive
60	CoV2	1	Wildtype D614G	29.9	29.2		37.0	0.0	0.0	0.0	0.0	28.8	SARS-CoV-2 positive
				Xpert® Xpress SARS-CoV-2									
				E-gene	N2-gene								
61	CoV2	4	Omicron	14.9	16.6		18.0	0.0	0.0	0.0	0.0	34.7	SARS-CoV-2 positive
63	CoV2	4	Omicron	22.7	24.7		24.5	0.0	0.0	0.0	0.0	34.3	SARS-CoV-2 positive
64	CoV2	4	Omicron	23.2	25.1		28.0	0.0	0.0	0.0	0.0	29.2	SARS-CoV-2 positive
62	CoV2	4	Omicron	27.1	28.6		22.4	0.0	0.0	0.0	0.0	37.0	SARS-CoV-2 positive
65	CoV2	4	Omicron	27.4	28.9		29.3	0.0	0.0	0.0	0.0	28.7	SARS-CoV-2 positive
				SeeGene SARS_CoV-2									
				RdRP	S-gene	N-gene							
73	CoV2	3	Delta	34.5	33.1	0.0	33.6	0.0	0.0	0.0	0.0	30.4	SARS-CoV-2 positive
77	CoV2	3	Beta	16.0	17.2	16.1	18.7	0.0	0.0	0.0	0.0	32.4	SARS-CoV-2 positive
69	CoV2	3	Delta	15.1	14.8	18.6	16.2	0.0	0.0	0.0	0.0	32.8	SARS-CoV-2 positive
79	CoV2	3	Beta	21.3	21.4	20.3	23.1	0.0	0.0	0.0	0.0	29.0	SARS-CoV-2 positive
74	CoV2	3	Delta	19.0	18.5	21.9	19.4	0.0	0.0	0.0	0.0	34.1	SARS-CoV-2 positive
71	CoV2	3	Delta	20.0	19.4	23.4	19.5	0.0	0.0	0.0	0.0	30.8	SARS-CoV-2 positive
75	CoV2	3	Delta	19.7	19.6	24.0	22.2	0.0	0.0	0.0	0.0	28.7	SARS-CoV-2 positive
66	CoV2	3	Delta	23.4	23.0	27.0	24.2	0.0	0.0	0.0	0.0	29.3	SARS-CoV-2 positive
78	CoV2	3	Beta	29.1	28.5	27.8	30.3	0.0	0.0	0.0	0.0	28.9	SARS-CoV-2 positive
76	CoV2	3	Alpha	20.1	19.7	27.9	21.2	0.0	0.0	0.0	0.0	31.1	SARS-CoV-2 positive
68	CoV2	3	Delta	26.1	26.2	28.8	27.9	0.0	0.0	0.0	0.0	28.4	SARS-CoV-2 positive
80	CoV2	3	Beta	30.1	30.4	29.8	33.6	0.0	0.0	0.0	0.0	29.0	SARS-CoV-2 positive
72	CoV2	3	Delta	32.6	32.5	33.9	34.1	0.0	0.0	0.0	0.0	30.7	SARS-CoV-2 positive
67	CoV2	3	Delta	32.2	30.7	34.5	31.7	0.0	0.0	0.0	0.0	30.0	SARS-CoV-2 positive
70	CoV2	3	Delta	31.1	29.9	34.6	30.9	0.0	0.0	0.0	0.0	29.1	SARS-CoV-2 positive
81	Neg	i2	SARS-CoV-2 negative	0.0	0.0		0.0	0.0	0.0	0.0	0.0	31.5	SARS-CoV-2 negative
82	Neg	i2	SARS-CoV-2 negative	0.0	0.0		38.5	0.0	0.0	0.0	0.0	33.2	SARS-CoV-2 positive
83	Neg	i2	SARS-CoV-2 negative	0.0	0.0		39.8	0.0	0.0	0.0	0.0	30.5	SARS-CoV-2 positive
84	Neg	i2	SARS-CoV-2 negative	0.0	0.0		0.0	0.0	0.0	0.0	0.0	29.8	SARS-CoV-2 negative
85	Neg	i2	SARS-CoV-2 negative	0.0	0.0		0.0	0.0	0.0	0.0	0.0	29.4	SARS-CoV-2 negative
86	Neg	i2	SARS-CoV-2 negative	0.0	0.0		0.0	0.0	0.0	0.0	0.0	31.4	SARS-CoV-2 negative
87	Neg	i2	SARS-CoV-2 negative	0.0	0.0		0.0	0.0	0.0	0.0	0.0	28.8	SARS-CoV-2 negative
88	Neg	i2	SARS-CoV-2 negative	0.0	0.0		0.0	0.0	0.0	0.0	0.0	30.8	SARS-CoV-2 negative
89	Neg	i2	SARS-CoV-2 negative	0.0	0.0		0.0	0.0	0.0	0.0	0.0	30.2	SARS-CoV-2 negative
90	Neg	i2	SARS-CoV-2 negative	0.0	0.0		0.0	0.0	0.0	0.0	0.0	29.6	SARS-CoV-2 negative
91	Neg	i2	SARS-CoV-2 negative	0.0	0.0		0.0	0.0	0.0	0.0	0.0	29.2	SARS-CoV-2 negative
92	Neg	i2	SARS-CoV-2 negative	0.0	0.0		0.0	0.0	0.0	0.0	0.0	32.0	SARS-CoV-2 negative

AccuPlex COV2/Flu A/Flu B/RSV Reference Material										
Concentration	Targets	6/10colour	CoV2	Flu A1	Flu A2	Flu B	RSV	SPC	Result	Comment
100cp/mL	COV2/Flu A/Flu B/RSV	6	38.7	37.5	0	35.8	38	28.9	COV2/FluA/FluB/RSV positive	FluA 2 neg
100cp/mL	COV2/Flu A/Flu B/RSV	10	38	37.7	38.6	35.1	38.2	28.7	COV2/FluA/FluB/RSV positive	
100cp/mL	COV2/Flu A/Flu B/RSV	10	38.2	38.4	38.7	35.4	39.7	29.5	COV2/FluA/FluB positive;	RSV negative; Ct >39
250cp/mL	COV2/Flu A/Flu B/RSV	6	37.1	36.1	37.3	35.1	37	29.5	COV2/FluA/FluB/RSV positive	
250cp/mL	COV2/Flu A/Flu B/RSV	10	37.2	37.3	40.6	34.4	36.8	28.9	COV2/FluA/FluB/RSV positive	FluA 2 neg
250cp/mL	COV2/Flu A/Flu B/RSV	6	37.1	37.4	38	34.6	37.1	28.7	COV2/FluA/FluB/RSV positive	
500cp/mL	COV2/Flu A/Flu B/RSV	10	36.1	35.9	36.5	33.5	35.9	28.8	COV2/FluA/FluB/RSV positive	
500cp/mL	COV2/Flu A/Flu B/RSV	6	35.7	35.6	36.8	33.4	35.7	28.6	COV2/FluA/FluB/RSV positive	
500cp/mL	COV2/Flu A/Flu B/RSV	10	36.3	35.9	36.8	33.6	35.7	29.5	COV2/FluA/FluB/RSV positive	
1000cp/mL	COV2/Flu A/Flu B/RSV	6	35.3	35.1	35.6	33.6	35.8	32	COV2/FluA/FluB/RSV positive	
1000cp/mL	COV2/Flu A/Flu B/RSV	10	35.2	35.2	35.7	32.7	35	30.5	COV2/FluA/FluB/RSV positive	
1000cp/mL	COV2/Flu A/Flu B/RSV	6	35.1	35	35.6	32.2	34.5	29.2	COV2/FluA/FluB/RSV positive	
5000cp/mL	COV2/FLuA/FluB/RSV	6	32.4	31.8	33.2	30.4	32.6	30.6	COV2/FluA/FluB/RSV positive	
500cp/mL	Alpha	6	35	0	0	0	0	28.9	SARS-CoV-2 positive	Flu A/Flu B/RSV negative
500cp/mL	Alpha	10	34.4	0	0	0	0	28.6	SARS-CoV-2 positive	Flu A/Flu B/RSV negative
500cp/mL	Beta	6	34.8	0	0	0	0	29.5	SARS-CoV-2 positive	Flu A/Flu B/RSV negative
500cp/mL	Beta	10	34.8	0	0	0	0	30.1	SARS-CoV-2 positive	Flu A/Flu B/RSV negative
500cp/mL	Gamma	6	34.8	0	0	0	0	28.8	SARS-CoV-2 positive	Flu A/Flu B/RSV negative
500cp/mL	Gamma	10	34.7	0	0	0	0	28.9	SARS-CoV-2 positive	Flu A/Flu B/RSV negative
500cp/mL	Delta	10	34.7	0	0	0	0	29.5	SARS-CoV-2 positive	Flu A/Flu B/RSV negative
500cp/mL	Delta	6	34.4	0	0	0	0	30	SARS-CoV-2 positive	Flu A/Flu B/RSV negative
